# Quality of life as a prognostic marker in pulmonary arterial hypertension

**DOI:** 10.1186/s12955-014-0130-3

**Published:** 2014-08-30

**Authors:** Caio JCS Fernandes, Barbara CS Martins, Carlos VP Jardim, Rozana M Ciconelli, Luciana K Morinaga, Ana Paula Breda, Susana Hoette, Rogério Souza

**Affiliations:** Pulmonary Department, Heart Institute – University of Sao Paulo Medical School, Medical School. Av. Dr. Eneas de Carvalho Aguiar, 44, 05403-000 Sao Paulo, Brazil; Rheumatology Department, Federal University of Sao Paulo, Sao Paulo, Brazil

**Keywords:** Quality of life, Pulmonary arterial hypertension, Survival, Prognosis, Treatment

## Abstract

**Background:**

Improvement in quality of life together with better survival are the ultimate goals in the treatment of pulmonary arterial hypertension (PAH) patients. The objective of this study was to evaluate the health-related quality of life (HRQL) of pulmonary arterial hypertension (PAH) patients with the SF-36 generic questionnaire and to identify the prognostic implication of this assessment.

**Methods:**

Fifty-four consecutive newly diagnosed PAH patients (WHO classification group I) in a single PAH reference center were included. Patients were evaluated at baseline for clinical and hemodynamic parameters, and they subsequently received first-line therapy with either an endothelin receptor antagonist or a phosphodiesterase-5 inhibitor. After 16 weeks of specific PAH therapy, all patients were re-evaluated using a 6MWT and a SF 36 questionnaire, and then they were followed up for at least 36 months.

**Results:**

After treatment, the patients demonstrated an improved 6MWT (414 ± 124 m vs. 440 ± 113 m, p = 0.001). Specific PAH therapy also improved the HRQL scores.

Patients with a baseline Physical Component Score (PCS) higher than 32 had a better survival rate than those who had a score under 32 (p = 0.04). Similarly, patients with a PCS of at least a 38 after the 16 week therapy period had a better survival rate when compared with those who did not achieve this value (p = 0.016). Unlike the absolute PCS values, the post-treatment PCS variability was unable to predict better survival rates (p = 0.58).

**Conclusions:**

Our findings suggest that HRQL is associated with prognosis in PAH. Furthermore, achieving pre-determined PCS scores might represent a specific goal to be reached in treatment-to-target strategies.

## Introduction

Pulmonary arterial hypertension (PAH) is a chronic disease caused by elevated pressure in the lung vasculature, eventually leading to the right ventricle failure and death due to circulatory collapse [1]. PAH is a rare condition with an annual incidence of 1.1 to 7.6 cases per million and a prevalence of 15 to 26 cases per million [2]. Consequently, the development of clinical trials with useful endpoints is challenging because mortality, as the primary endpoint, is usually not feasible. Therefore, alternative methods for evaluating the clinical responses to specific PAH therapies were widely used in the design of randomized, controlled trials and in clinical practice [3].

Studies that addressed targeted PAH therapies, such as prostanoids [4], endothelin receptors antagonists (ERA) [5], and phosphodiesterase-5 inhibitors (PDE5i) [6], have demonstrated that several parameters, including the New York Heart Association functional class assessment, the six-minute walk test (6MWT), serum biomarkers (BNP and NT-ProBNP) [7-9] and invasive hemodynamic (Cardiac Index, Mean Pulmonary Artery Pressure and Right Atrium Pressure) measurements, closely associate with disease severity and are directly improved by medical treatments suggesting a potentially positive influence of these therapies on PAH patients’ mortality [10].

Nevertheless, the main objective of specific therapies for PAH patients was to improve not only the quantity of life, or survival, but also the quality of life. PAH patients are well known to have a severely impaired health-related quality of life (HRQL) [11]. Reports of evaluation tools that specifically address the impaired HRQL induced by PAH are still limited in the literature [12,13] and are not routinely employed in general practice in PAH patients. The development of PAH treatment strategies that could simultaneously evaluate both the quantity and quality of life would be potentially useful in the clinical management of this disease.

Quality of life can be evaluated in several chronic diseases using specific and validated inquiries such as the Short-Form 36 Health Survey (SF-36) [14]. This highly reproducible, well-known, non-invasive and frequently used survey, for such evaluations, is widely available in several different languages [15]. Although specific PAH therapies have already been demonstrated to improve HRQL [16-19], the association of this improvement and survival has not been properly addressed.

The aim of this study was to evaluate the impact of targeted PAH therapies on the HRQL using the SF-36 and addressing HRQL association to survival.

## Methods

### Patient population

Newly diagnosed PAH patients (group I of the Dana Point Classification system) [20] followed in a large PAH reference center (Heart Institute) in São Paulo, Brazil, were included in this study. The protocol was approved by the Institutional Review Board.

The baseline patient evaluations included obtaining information on demographics and medical histories and included the New York Heart Association (NYHA) functional class assessments, physical examinations and routine laboratory tests. Data from 6MWT assessments [21], quality of life questionnaires [19] and right heart catheterizations using standard techniques were also obtained [22].

The PAH patients’ quality of life was assessed using the SF 36 questionnaire, which consists of 36 questions covering eight domains. Four domains are projected to reflect the impact of the disease on the physical component of the patients life (Physical Component Score – PCS), and the four remaining domains are projected to reflect the impairment caused by the disease on mental status (Mental Component Score – MCS). The scores were converted to a 100-points score scale where higher scores indicated a better QOL. The SF-36 questionnaire was given by a person trained to use the QOL assessment tool [15] and was chosen because it had been validated for use in the native language of the patients who comprised the study [23].

Patients with PAH are defined as having a mean pulmonary artery pressure (mPAP) > 25 mmHg with a normal pulmonary artery occlusion pressure (PAOP <15 mmHg) [20]. Patients were classified as having idiopathic pulmonary arterial hypertension (IPAH) when PAH was diagnosed in the absence of chronic thromboembolic pulmonary hypertension and was not associated with significant left heart or lung disease or with other known conditions, including HIV infection, portal hypertension, congenital systemic-pulmonary shunts, connective tissue diseases and exposure to appetite suppressants. Patients were classified as having schistosomiasis-associated PAH (Sch-PAH) when the presence of PAH was associated with liver ultrasonographic observations that were highly suggestive of mansonic schistosomiasis (left lobe enlargement and/or periportal fibrosis). Additionally, the patients must demonstrate at least one of the following features: 1) the endemic regional exposure to schistosomiasis, 2) previous treatment for schistosomiasis and 3) the presence of *Schistosoma mansoni* eggs in the stool following examinations or rectal biopsies [24,25].

### Treatments

In the absence of any contraindication (e.g., high risk of gastrointestinal bleeding or presence of esophageal varices) the patients received oral anticoagulation; diuretics and oxygen were prescribed as needed.

Patients received a PAH first-line therapy of either an ERA or a PDE5i. The choice between the agents was based on the drug availability at our facility. After 16 weeks of specific PAH therapy, all patients were re-evaluated by means of the 6MWT and the SF 36 HRQL questionnaire. Patients were then followed up for up to 36 months for survival analysis.

### Statistical analysis

Analysis was performed using the SPSS 15 statistical package (SPSS, Inc., Chicago, IL). All continuous variables are expressed as median ± SD, whereas the categorical data are presented as proportions. The Cronbach’s α coefficient was calculated for all SF 36 scores to verify the questionnaire reproducibility and reliability, and values higher than 0.7 were considered satisfactory.

To compare the baseline and post-treatment characteristics, a paired t-test was used. For survival analysis, the first hemodynamic evaluation was considered to be the date of diagnosis. All-cause mortality was used because of the lack of information about the specific cause of death in several cases. No patients were lost to follow-up during the study period. The patient survival was estimated using the Kaplan-Meier method, and the log-rank test was applied to compare these curves. A p-value of less than 0.05 was considered as statistically significant.

## Results

The study population consisted of 54 PAH patients. The baseline clinical, functional and hemodynamic data are shown in Table 1 and are compatible to previously published series for PAH patients.

**Table 1:** **Baseline clinical and hemodynamic data (n = 54)**

**Age (years)**	**44 ± 12**
Sex (f/m)	45/9
Etiology n (%)	
Idiopathic	37 (68)
Associated to	
Schistosomiasis	7 (13)
CTD	8 (15)
CHD	2 (4)
NYHA functional class n (%)	
II	14 (26)
III	28 (52)
IV	12 (22)
6MWT (m)	418 ± 121
mPAP (mmHg)	64 ± 17
PAOP (mmHg)	10 ± 2
Cardiac output (L/min)	4 ± 1
PVR (WU)	15 ± 8
Therapy n (%)	
ERA	46 (85)
PDE-5 inhibitors	8 (15)

*CTD* Connective tissue disease, *CHD* Congenital heart disease, *NYHA* New York Heart Association, *6MWT* Length in non-encouraged six-minute-walk test, *RAP* Right Atrial Pressure, *mPAP* Mean Pulmonary Artery Pressure, *PAOP* Pulmonary Artery Occlusion Pressure, *PVR* Pulmonary Vascular Resistance, *ERA* Endothelin Receptor Antagonists, *PDE-5* Phosphodiesterase-5 inhibitor.

**Figure 1:**
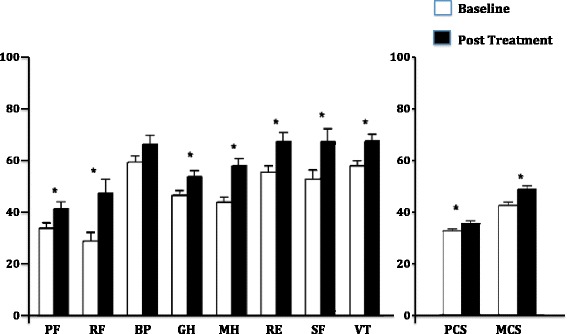
SF-36 score domains variations after specific PAH therapy for 16 weeks (* p < 0.05). PF – Physical Function, RF – Role Physical, BP – Bodily Pain, GH – General Health, MH – Mental Health, RE – Role Emotional, SF – Social Function, VT – Vitality, PCS – Physical Component Score, MCS – Mental Component Score.

After the 16-week treatment period using an ERA (in 85% of all cases) or a PDE5i (in the remaining 15%), patients demonstrated a significantly improved 6MWT (414 ± 124 m vs. 440 ± 113 m, p = 0.001) as well as in functional class (p = 0.02). Specific PAH therapy also improved the HRQL scores as evaluated by the SF 36 for all the domains except for bodily pain (Figure 1). The Cronbach’s α coefficient was found to be 0.852 and considered adequate. Interestingly, baseline PCS correlated with 6MWD (r = 0.493, p < 0.01) and also with functional class (r = -0.576, p < 0.01).

**Figure 2:**
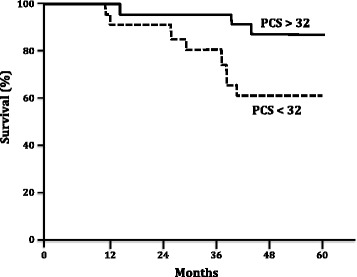
**Kaplan Meier survival rates according baseline SF-36 Physical Component Score (PCS).** Log rank test, p = 0.04.

**Figure 3:**
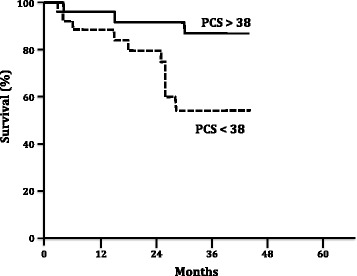
**Kaplan Meier survival rates according SF-36 Physical Component Score (PCS) after 16 weeks of specific PAH therapy.** Log rank test, p = 0.016.

**Figure 4:**
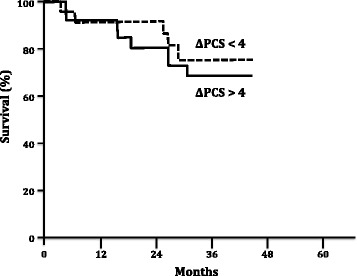
**Kaplan Meier survival rates according variation of SF-36 Physical Component Score (PCS) after 16 weeks of specific PAH therapy.** Log rank test, p = 0.58.

Furthermore, the HRQL scores were able to discriminate between patients with a better survival both in the baseline and in the post-treatment periods. Patients with a baseline PCS higher than the median value (32) had a significantly improved survival (p = 0.04, Figure 2). A similar pattern was found in the post-treatment evaluation; patients who reached more than the median post-treatment PCS value (38) had a better survival rate when compared to those who did not reach this threshold (p = 0.016, Figure 3). Notably, the absolute change in PCS after treatment was not predictor of survival in this study (p = 0.58, Figure 4).

## Discussion

This study demonstrated the association of HRQL assessment, more specifically, the use of the SF 36 questionnaire, with prognosis in PAH. The baseline HRQL measurements can distinguish a high-risk subgroup of PAH patients with a poor survival outlook. Furthermore, HRQL might also represent a viable treatment goal because reaching a specific post-treatment threshold on the PCS was associated with better survival.

The aim of specific PAH therapy is to improve patient survival. However, despite all the recent advances in diagnostic and therapeutic modalities for PAH, the mortality rate remains high, with a 5-year survival of 57% [26]. Despite the need for improved treatment strategies, these data also reinforce the significance of improving the HRQL for such a relentless and progressive disease. Our study attempts to concatenate both survival and quality of life evaluations as a result of specific drug therapy. While attempting to improve the HRQL and establishing harder endpoints for clinical therapy, it is possible that better survival rates might also be achieved.

Previous studies have already demonstrated that the HRQL of PAH patients is severely reduced when compared to healthy individuals [11]. PAH patients present with reduced physical mobility, significant dyspnea and increased difficulties in social interactions. Some trials involving PAH patients, when evaluating the response to different therapies, used HRQL questionnaires as secondary endpoints but reached variable results. One study showed that the HRQL improved modestly after bosentan therapy and this improvement was stable for 3 months [16]. The same response pattern was also described with another ERA, sitaxsentan [27]. However, the HRQL remained unchanged in other clinical trials using therapeutic drugs known to be effective for PAH patients such as treprostinil [4] and ambrisentan [28]. Nevertheless, recent systematic review on the effect of targeted therapies in HRQL of PAH patients demonstrated that HRQL consistently improved after specific intervention, even considering the heterogeneity of the instruments used for quality of life evaluation among the different studies [29]. In our study, except for bodily pain, all domains significantly improved after treatment with specific PAH therapies, resulting also in a significant improvement in the physical component score as in the mental component score.

Recently, Roman *et al*. described the HRQL of PAH and chronic thromboembolic pulmonary hypertension (CTEPH) Spanish cohorts and described similar baseline results to those in our study [30]. In the Roman et al. study, both the SF-36 and EuroQol 5D questionnaires were able to identify the patients with a worse prognosis. Nevertheless, our study is the first to demonstrate that the clinical response to specific PAH therapy could be effectively measured using a SF-36 questionnaire. In our study, the baseline physical score component and the PCS after 16 weeks of specific PAH treatment was associated with patient survival. In a pattern similar to one previously described for the 6MWT [31], the absolute variation of PCS after treatment was not associated with survival suggesting that achieving a specific PCS threshold is more important than improving the PCS itself to determine a better prognosis. This is the first time that a specific goal associated to HRQL is described in addition to others previously defined in the literature [32].

For 6MWT, it has been previously suggested that improvements of 33 m would represent the minimal important difference (MID) to reflect improvements in the quality of life of PAH patients [17]. A more recent analysis of the raw data from ten different trials tried to establish the relationship between the changes in 6MWD and the probability of clinical deterioration to occur [33]. In this study, Gabler et al. demonstrated that on average 6MWD improved by 22.4 m with active treatment; however, this improvement accounted only for a small proportion of the treatment effect, suggesting the change in 6MWT would not represent a valid surrogate in PAH, confirming the previous findings from Sitbon et al. [31]. These studies, in line with our results, suggested that a minimum functional status should be reached in order to reflect better prognosis.

A MID in SF-36 scores has been suggested for PAH. Gilbert et al. mathematically determined the MID, throughout distribution-based models, suggesting MID of 13 to 25, depending on the domain of the SF-36 [34]. No analysis on the physical or mental component scores were reported. The same study suggested a MID of 41m for the 6MWT. However, the analysis was based solely on the distribution of the results, since the authors had no surrogate marker to use as an anchor to determine the relevance of the MID, thus preventing the extrapolation of the results; differently, our study used survival as a hard endpoint for the analysis of the minimum threshold in HRQL, more specifically in PCS, that could reflect better long-term survival in PAH.

In 2006 McKenna *et al* developed a HRQL scale specifically for the PAH population using the Cambridge Pulmonary Hypertension Outcome Review (CAMPHOR) [13]. The CAMPHOR questionnaire comprises overall symptoms (comprising Energy, Breathlessness and Mood subscales), functionality and HRQL, and demonstrated good values for the Cronbach’s α coefficient, and reproducibility. Recently, several studies used the CAMPHOR scale in several cohorts across the world and validated the questionnaire in German [35], French-Canadian [36] and Swedish [37] languages. The CAMPHOR questionnaire was also applied to different English-speaking PAH patient populations such as American [38], Canadian [36], Australian and New Zealander [39] with good results. However, the predictive value of CAMPHOR appears to be restricted to baseline evaluations. Recently, McCabe *et al* demonstrated that repeated CAMPHOR assessments over time did not add any predictive value for clinical deterioration to that obtained at diagnosis in an IPAH population followed for 8 years [40]. These data limit the prospective utility of CAMPHOR assessments for clinical follow-up.

This study has limitations that need to be acknowledged. First, it was a single center study in a country with a peculiar distribution of PAH etiologies where schistosomiasis-associated pulmonary arterial hypertension holds high epidemiologic relevance [41-43]. Although our center is the largest reference center in the country, the potential selection bias associated with this limitation should be taken into consideration when extrapolating our results. Furthermore, we decided not to use a health-related quality of life questionnaire specific for PAH, but a general one, projected to evaluate chronic diseases. Nevertheless, the SF-36 is the best-known and studied HRQL questionnaire, worldwide; additionally, it is rapid, easy and reliable.

Despite these limitations, our results support the association of PAH prognosis with general HRQL evaluation with the SF-36 questionnaire, at baseline and after specific PAH therapies. Moreover, achieving pre-determined PCS scores might represent a specific goal to be reached in treatment-to-target strategies, as currently is recommended for PAH.
